# Draft Genome Sequence of the 2-Chloro-4-Nitrophenol-Degrading Bacterium *Arthrobacter* sp. Strain SJCon

**DOI:** 10.1128/genomeA.00058-13

**Published:** 2013-03-07

**Authors:** Surendra Vikram, Shailesh Kumar, Bhumika Vaidya, Anil Kumar Pinnaka, Gajendra Pal Singh Raghava

**Affiliations:** a Microbial Type Culture Collection and Gene Bank (MTCC), Institute of Microbial Technology (IMTECH), Chandigarh, India; b Bioinformatics Centre, CSIR-Institute of Microbial Technology, Chandigarh, India

## Abstract

We report the 4.39-Mb draft genome sequence of the 2-chloro-4-nitrophenol-degrading bacterium *Arthrobacter* sp. strain SJCon, isolated from a pesticide-contaminated site. The draft genome sequence of strain SJCon will be helpful in studying the genetic pathways involved in the degradation of several aromatic compounds.

## GENOME ANNOUNCEMENT

*Arthrobacter* spp. are widely distributed in the environment, and members of this genus have been found to be involved in various metabolic activities, like chromium reduction (1), biodegradation of 4-nitrophenol, 4-chlorophenol (2), and 4-fluorocinnamic acid (3), and biotransformation of benzene and toluene (4). *Arthrobacter* sp. strain SJCon was isolated from a pesticide-contaminated site in Punjab, India, by an enrichment method. The strain SJCon can degrade nitroaromatic compounds, such as *p*-nitrophenol and 2-chloro-4-nitrophenol, as sole sources of carbon and energy (5). Whole-genome sequencing of *Arthrobacter* sp. SJCon was done using Ion torrent technology at CSIR-IMTECH and produced a total of 2,234,522 reads of average length 147.6 nucleotides (nt), with a coverage of 73.3× of a 4.5-Mb genome (expected size). All reads have been assembled by using Newbler version 2.5.3 (Roche) at the default parameters. A total of 142 contigs of >500 bp length were constructed, with an N_50_ of 63.6 kb; the largest contig assembled measured 143.0 kb. The final genome draft, which consists of 4,389,620 bp, has been used for genome annotation by the RAST (Rapid Annotations using Subsystems Technology) system (6), RNAmmer 1.2 (7), and ARAGORN software (8). A total of 4,242 coding sequences (CDSs), 3 rRNAs, and 54 tRNAs were predicted. We have also reconfirmed the 16S rRNA gene sequence by Sanger’s sequencing and found 100% identity with the RNAmmer-predicted 16S rRNA sequence. However, the 16S rRNA sequence of strain SJCon, available at the GenBank database (accession no. GQ927310.2), showed only 98.94% identity with the 16S rRNA gene sequence from this annotated genome, which might be due to a discrepancy in the previous gene sequence submission. RAST annotation shows that *Arthrobacter chlorophenolicus* A6 (score 542), *Arthrobacter* sp. FB24 (score 537), and *Arthrobacter aurescens* TC1 (score 477) are the closest neighbors of the strain SJCon. This annotation indicates that the genes of glycolysis and gluconeogenesis, galactose metabolism, tricarboxylic acid (TCA) cycle, propanoate metabolism, and the pentose phosphate pathway are present in the genome of strain SJCon. In the annotation, we found the genes of flavin adenine dinucleotide (FAD)-binding monooxygenase, nitrilotriacetate monooxygenase component B (EC 1.14.13.-), 4-hydroxyphenylacetate 3-monooxygenase (EC 1.14.13.3), cyclohexanone monooxygenase (EC 1.14.13.22), catechol 2,3-dioxygenase (EC 1.13.11.2), protocatechuate 3,4-dioxygenase alpha and beta chain (EC 1.13.11.3) and catechol 1,2-dioxygenase (EC 1.13.11.1), 2-keto-4-pentenoate hydratase (EC 4.2.1.80), 3-oxoadipate coenzyme A (CoA)-transferase subunit A and B (EC 2.8.3.6), and 3-ketoacyl-CoA thiolase (EC 2.3.1.16) to be involved in the catabolism of aromatic hydrocarbons.

### Nucleotide sequence accession numbers.

This Whole Genome Shotgun project has been deposited at DDBJ/EMBL/GenBank under the accession no. AOFD00000000. The version described in this paper is the first version, AOFD01000000.
